# Ward-level risk factors associated with nosocomial coronavirus disease 2019 (COVID-19) outbreaks: A matched case–control study

**DOI:** 10.1017/ash.2022.11

**Published:** 2022-03-25

**Authors:** Reto Thoma, Philipp Kohler, Sabine Haller, Jasmin Maenner, Matthias Schlegel, Domenica Flury

**Affiliations:** Departement of Infectious Diseases and Hospital Epidemiology, Cantonal Hospital of St. Gallen, St. Gallen, Switzerland

The coronavirus disease 2019 (COVID-19) pandemic made its way from private, public, and work spaces into healthcare systems all over the world, imposing an unexpected burden on global health care. COVID-19 outbreaks in acute-care hospitals are a frequent problem, increasing morbidity and mortality among patients and leading to staff shortages.^1,2^ Prevention of nosocomial severe acute respiratory syndrome coronavirus-2 (SARS-CoV-2) transmission in acute-care hospitals represents a major challenge. One important reason is that diagnosis in asymptomatic or presymptomatic patients and healthcare workers (HCWs) is often delayed.^3^ Also, understaffing during the pandemic may decrease adherence to standard hygiene measures.^4^ Before the SARS-CoV-2 vaccine became available, we observed several wards with nosocomial COVID-19 outbreaks, whereas others were spared. We sought to identify ward-level risk factors associated with nosocomial COVID-19 outbreaks.

## Methods

**Fig. 1.**
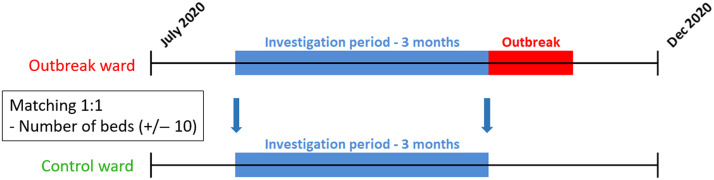
**Comparison of case and control wards.** Between July and December 2020, 9 Outbreak and 9 control wards were matched for appropriate number of beds. The same period of investigation was chosen retrospectively for outbreak and control wards.

We conducted a matched case–control study in our 700-bed tertiary-care center during the second wave of the COVID-19 pandemic. Infection prevention and control (IPC) measures consisted of the use of personal protective equipment (PPE), social distancing, placing confirmed cases in cohorts, and visitor restrictions. Routine testing of asymptomatic HCWs was not implemented. We defined nosocomial SARS-CoV-2 outbreaks as the occurrence of ≥2 patients with nosocomial infection within a 14-day period on the same ward. Nosocomial infection was defined as a positive SARS-CoV-2 test on day 5 or later of hospital admission. Wards with at least 1 nosocomial outbreak between July and December 2020 were defined as outbreak wards. Wards without outbreaks served as controls. Intensive care units and designated COVID-19 wards were excluded. Ward matching was done 1:1 for approximate number of beds (±10) and time of the outbreak by choosing the same period of investigation on control and outbreak wards. The beginning of an outbreak was defined as the day of the first positively tested nosocomial COVID-19 patient on the ward; the same day was chosen as reference date for the control ward (Fig. 1). For the 3 months prior to the start of the outbreak, we retrospectively collected data on ward-level factors: structural data (ie, number of beds and bed occupancy preceding ward outbreak), aggregated patient data (ie, age, length of stay, number of entries per bed, % of COVID-19 entries and workload allocated to complicated patients), and aggregated staff-specific data (ie, age, sex ratio, years on duty in our center as a measure of work experience, workload, number of nurses per 100 care days, degree of employment, number of HCWs who tested positive within 2 weeks before until 2 days after beginning of the outbreak or reference date and within 3 months to 2 weeks preceding the same date). We performed univariable analyses using the paired Wilcoxon signed-rank test. *P* ≤ .05 was considered statistically significant. We used SPSS Statistics for Windows version 20.0 software for all statistical analyses (IBM, Armonk, NY).

## Results

We observed 9 outbreak wards during the study period: 3 surgical and 6 medical wards. The median number of beds was 25 (range, 17–31), and the median number of nurses was 30 (range, 19–41). In total, 40 patients acquired nosocomial COVID-19, with a median of 4 patients per outbreak ward (range, 2–7). Compared to control wards, outbreak wards demonstrated trends toward higher numbers of beds per room (2.22 vs 1.97; *P* = .09) and younger ages of nurses (33.3 vs 36.2 years; *P* = .17). The only factor that was significantly different between outbreak and control wards was the percentage of positively tested HCWs immediately before the outbreak or reference date (9.7% vs 2.7%; *P* = .04). We detected no difference in the percentage of infected HCWs in the earlier period (prior to 2 weeks before the outbreak). We detected no association with factors reflecting structural characteristics, workload, patient turnover, or work experience (Table 1).

**Table 1.** Investigated Ward-Level Risk Factors for COVID-19 Ward Outbreaks^a^

Variable	Outbreak Wards, Mean (SD)	Control Wards, Mean (SD)	P Value
**Structural data**			
No. of beds per room	2.22 (0.4)	1.97 (0.6)	.088
% bed occupancy preceding ward outbreak^b^	92.3 (5.3)	92.3 (4.6)	.374
**Aggregated patient data**			
Patient age	62.8 (6.3)	59.8 (7.6)	.260
Length of hospital stay	3.1 (1.3)	4.4 (2.5)	.396
No. of entries per bed	6.1 (3.6)	6.1 (2.8)	.859
% COVID-19 entries^c^	2.3 (3.4)	1.4 (2.6)	.223
% care days invested for complicated patients^d^	2.6 (1.7)	1.8 (1.0)	.441
**Aggregated staff-specific data** ^e^			
Age	33.3 (3.1)	36.2 (5.2)	.172
Sex, % female	95.0 (3.3)	94.8 (3.8)	.671
Years on duty^f^	7.8 (2.2)	7.7 (2.7)	1.000
No. of nurses per 100 care days^g^	1.52 (0.2)	1.47 (0.4)	.766
Degree of employment per nurse (%)	80.6 (5.4)	78.5 (4.5)	.139
Objective workload nurses^h^	4.6 (0.7)	4.3 (0.8)	.326
Subjective workload nurses^i^	4.8 (0.3)	4.3 (0.4)	.231
% of COVID-19 positive HCWs 14 d preceding ward outbreak^j,k^	9.7 (9.8)	2.7 (3.9)	.036
% of COVID-19 positive HCWs 3 mo to 14 d preceding ward outbreak^k^	4.4 (6.0)	4.7 (5.8)	.866

Note. COVID-19, coronavirus disease 2019, HCW, healthcare worker, LEP, Leistungserfassung in der Pflege.

a Unless differently specified, data of outbreak and control wards reflect a period of 3 months preceding the outbreak.

b In entire months, outbreak month included if the outbreak started after the 20th of the month.

c % of total entries in the period.

d % of total care days, complicated patients correspond to patients with a LEP nursing category 7 or higher (ie, nursing demand of 720 minutes per 24 hours or more).

e Nurses unless differently specified.

f In our center.

g Care day is any day on which nursing was registered for a patient.

h Measured as objective LEP score.

i Measured as subjective LEP score, corresponds to subjective daily workload as indicated by nurses (scale 1–7).

j Period corresponds to 14 days prior to 2 days after detection of the first nosocomial case of the outbreak.

k Including nurses, therapists, and doctors.

## Discussion

In this matched case–control study, the only factor associated with occurrence of nosocomial COVID-19 outbreaks was an increased number of infected HCWs shortly before the outbreak.

This finding supports the hypothesis that infected HCWs are an important source of nosocomial COVID-19.

Our data agree with those of other studies. In a recent investigation of nosocomial COVID-19 outbreaks using whole-genome sequencing, the investigators concluded that SARS-CoV-2 transmission occurred most likely from HCW to patients.^5^ Because affected patients were highly dependent on nursing care, close contact between HCWs and patients was hypothesized to be the driving force for transmission. Similar findings have been reported by others.^6^ Our study, using a different approach by comparing ward-level factors between outbreak and non-outbreak wards, adds to the mounting evidence that HCWs are indeed the main source of nosocomial COVID-19 among patients. These findings corroborate the importance of adherence to IPC measures, not only in hospitals but also in public and private environments. Furthermore, vaccination or repetitive testing of asymptomatic HCWs might be other useful measures to efficiently prevent nosocomial SARS-CoV-2 infection.

Our study has limitations. We lacked whole-genome sequencing data for SARS-CoV-2 isolates. Also, we were not able to perform multivariable analyses because of the limited sample size.

In conclusion, our data support findings from other studies suggesting that infected HCWs are the main source of nosocomial COVID-19 cases, at least in a prevaccination era. Further studies will be needed to conclusively determine whether vaccination of HCWs and patients will reduce the rate of nosocomial COVID-19.
